# George H. Witter

**Published:** 1913-04

**Authors:** 


George H. Witter, P. & S. of Baltimore, 1885, died at his home in Wellsville, February 12, aged 59.

